# Review of risk factors and surgical treatment progress for gallbladder cancer

**DOI:** 10.1186/s41065-025-00608-z

**Published:** 2025-11-21

**Authors:** Jianguo Feng, Haihong Zhu, Xiaosong Wang

**Affiliations:** 1 Clinical Medical College, Qinghai University, Xining, Qinghai China; 2 Department of Hepatobiliary Surgery, Qinghai Provincial People’s Hospital, No.2 Gonghe Road, Xining, 810000 Qinghai China

**Keywords:** Gallbladder cancer, Risk factors, Surgical treatment, Molecular targeted therapy, Immunotherapy

## Abstract

**Objective:**

To systematically review the latest evidence on risk factors and surgical treatment for gallbladder cancer (GBC), with a focus on current controversies and consensus in international guidelines, analyze the application prospects of minimally invasive surgery in advanced GBC, and provide direction for clinical practice and future research.

**Methods:**

Literature on GBC risk factors, molecular mechanisms, and treatment strategies published from 2018 to 2024 was retrieved from databases including PubMed, Web of Science, and CNKI. The retrieved literature was summarized, compared, and critically analyzed.

**Results:**

The pathogenesis of GBC involves a combination of genetic, environmental, and metabolic factors. Beyond gallstones and polyps, mutations in TP53 and ERBB2/ERBB3 genes, metabolic syndrome (obesity, hyperglycemia, hyperlipidemia), and chronic infections (Salmonella, Helicobacter) are significant risk factors. Surgical resection remains the primary curative approach, yet the optimal extent of surgery is debated: Is hepatic resection always necessary for T1b stage? What is the oncological safety of laparoscopic surgery for T2 stage? What is the value of extended resection for T4 stage? Recently, targeted therapies (e.g., against ERBB2, NTRK) and mmune checkpoint inhibitors (anti-PD-1/PD-L1)have shown promise in advanced GBC.

**Conclusion:**

Combating GBC requires a comprehensive strategy encompassing health education, screening of high-risk populations, precise staging, and individualized multimodal treatment. Future research should focus on building molecular subtype-based prognostic models, conducting high-level clinical studies to resolve surgical controversies, and exploring the integration of novel adjuvant therapies with traditional surgery.

## Introduction

Gallbladder cancer (GBC) is the most common malignant tumor of the biliary tract system, accounting for 80%−95% of biliary malignancies [1]. Its onset is insidious, making early diagnosis challenging. Most patients are diagnosed at an advanced stage, exhibiting high malignancy and extremely poor prognosis, with a 5-year survival rate below 5% [2]. Surgery remains the only potentially curative approach, yet resection rates are low and postoperative recurrence is common. Recent advances in molecular biology and surgical techniques have yielded new insights into the etiology and treatment strategies for GBC. This paper aims to systematically review the risk factors and surgical treatment advances for GBC, focusing on comparing discrepancies in guideline recommendations, analyzing core controversies in the current surgical field, and exploring new opportunities presented by targeted and immunotherapy in the era of molecular subtyping. It seeks to provide more critical and forward-looking reference for clinical research and practice.

## Risk factors

The occurrence and development of gallbladder cancer (GBC) is a complex process involving multiple interacting factors, primarily including genetic susceptibility, alterations in the gallbladder microenvironment, and systemic metabolic status (Table 1).

**Table 1:** Categories of risk factors

Risk factor category	Specific factors	Main mechanisms	Clinical significance and relative risk of occurrence
Benign disease of gallbladder	Gallbladder stones	Long-term physical stimulation of gallstones leads to chronic injury, inflammation and epithelial atypical hyperplasia of gallbladder mucosa, and then canceration.	The most important risk factor. About 85% of patients with gallbladder cancer are complicated with gallstones, and the risk of gallstones is 13.7 times that of people without stones. If the diameter of the stone is more than 3 centimeters, the risk is 10 times that of less than 1 centimeter.
	Gallbladder polyps	Some polyp types (such as adenoma) are precancerous lesions, and dysplasia may occur. Enlarged polyps usually mean active cell proliferation and increased risk of malignant transformation.	Polyps ≥ 10 mm in diameter have a significantly increased risk of malignant transformation. Even if the polyp is small, the risk is increased if it is accompanied by stones, grows rapidly (> 3 mm/6 months) or is solitary/sessile, which is an important indication for surgical resection.
	Chronic cholecystitis and “porcelain gallbladder”	Chronic inflammatory environment produces a large number of inflammatory cytokines and reactive oxygen species, resulting in DNA damage. The “porcelain gallbladder” formed by calcification of gallbladder wall is highly correlated with canceration.	Chronic inflammation itself is considered to be precancerous. “Porcelain gallbladder” is highly correlated with gallbladder cancer and is a clear indication for preventive cholecystectomy.
	Anomalous pancreaticobiliary duct confluence	Congenital anatomic malformation leads to pancreatic juice reflux to the gallbladder, and the activated pancreatic enzymes produce long-term chemical stimulation to the gallbladder mucosa, causing repeated mucosal repair and malignant transformation.	It is a definite risk factor, and prophylactic surgery is a reasonable choice for patients with gallbladder mass.
Infection and biological factors	Biliary tract infection (Salmonella/Helicobacter pylori)	Chronic bacterial infection results in a persistent inflammatory response, and bacterial metabolites such as β-glucuronidase may induce bile acid degradation and produce carcinogens.	Salmonella typhi and Helicobacter pylori carriers are associated with an increased risk of GBC and have important epidemiological implications in certain populations.
Metabolic factors	Obesity	Insulin resistance, inflammation	All were associated with a significantly increased risk of GBC
	Type 2 diabetes	Hyperglycemic toxicity, cholestasis	
	Hypertriglyceridemia	Changes in bile composition	
Genetics and family history	Family history of gallbladder cancer or gallstones and mutations in certain genes (e.g. TP53, ERBB2)	Those with a family history of gallbladder cancer or gallstones have an increased risk of morbidity. Mutations in certain genes (e.g., TP53, KRAS) are common in gallbladder cancer and are associated with morbidity and prognosis.	Potential targets for prognosis prediction and targeted therapy

### Genetic factors and molecular alterations

Gallbladder carcinoma (GBC) exhibits significant molecular heterogeneity. Whole-exome sequencing studies have identified key driver gene mutations. TP53 is the most frequently mutated gene, with loss of function observed in over 40% of GBC cases [3]. This loss of function leads to uncontrolled cell cycle progression, impaired DNA damage repair, metabolic reprogramming, and evasion of apoptosis, thereby driving tumorigenesis [4]– [5]. Thus, in clinical-pathological diagnosis, complete loss or overexpression of p53 indicates high malignancy in gallbladder cancer. Postoperative detection of TP53 mutations suggests high disease recurrence risk, facilitating adjuvant therapy [6]. Studies indicate TP53 mutations impair apoptosis, rendering traditional chemotherapy targeting p53 pathway-mediated cancer cell death ineffective. Cutting-edge research [7] suggests WEE1 tyrosine kinase inhibitors effectively target TP53-mutated cancer cells via a “synergistic lethality” mechanism. Thus, actively exploring novel mechanism drugs like WEE1 inhibitors holds broad promise for gallbladder cancer diagnosis and treatment. Amplification or mutation of Erb-B2 receptor tyrosine kinase 2 (ERBB2) occurs in approximately 7–8% of GBC cases. It not only promotes tumor proliferation but also activates the PI3K/Akt pathway to upregulate PD-L1 expression in tumor cells, inducing immune escape. This makes ERBB2 a potential predictive biomarker for immunotherapy. Liu et al. [8] identified a novel exon-derived isoform originating from intron 14, termed ERBB2 i14e. This isoform confers trastuzumab resistance in cancer cells, though this therapeutic target requires further investigation. Other recurrent mutations include PIK3CA, CDKN2A/B, ARID1A, and KRAS [9]. These molecular alterations not only serve as oncogenic drivers but are increasingly emerging as potential targets for prognostic prediction and targeted therapy.

### Gallbladder microenvironment and infectious factors

Chronic inflammation serves as the common ground for the development of gallbladder cancer (GBC). Gallbladder stones represent the primary risk factor for GBC, with approximately 85%−90% of GBC patients presenting concurrent gallbladder stones. Individuals with gallstones face a 13.7-fold increased risk of developing the disease compared to those without stones. Furthermore, stones exceeding 3 cm in diameter carry a 10-fold higher risk than those smaller than 1 cm [10]. The underlying mechanisms may include prolonged physical irritation, bile stasis, and the resulting chronic cholecystitis, leading the mucosal epithelium through a progression of “metaplasia-dysplasia-carcinogenesis” [3]. Furthermore, specific pathogen infections play a crucial role. Carriers of Salmonella typhi exhibit a significantly increased risk of GBC [12]. The carcinogenic mechanism may involve DNA damage caused by secreted cytotoxic proteins (e.g., typhoid toxin) and biofilm formation that promotes chronic infection and stone formation [11]– [12]. Infections with Helicobacter pylori (H. pylori) and Helicobacter bilis4 (H. bilis4) have also been associated with elevated GBC risk [13]. These pathogens may induce carcinogenesis through local inflammatory responses and oxidative stress, though their colonization mechanisms within the gallbladder require further investigation.

### Metabolic syndrome and its components​

Metabolic syndrome as a whole is positively correlated with GBC risk. The effects of its individual components are as follows: Overweight and obesity: Studies indicate an increasing incidence of gallbladder cancer associated with high body mass index (BMI) [14]. The mechanisms by which obesity contributes to cancer remain unclear, but research [15] suggests insulin resistance, hyperinsulinemia, and insulin-like growth factor (IGF) may be implicated in obesity-related carcinogenesis. During insulin resistance, insulin’s inhibitory effect on lipolysis is suppressed, leading to increased release of free fatty acids (FFAs). Elevated FFAs exert a negative feedback effect on hepatic insulin uptake, causing insulin levels to rise. which in turn affects adipose-derived hormones including leptin, adiponectin, resistin, and tumor necrosis factor (TNF-α). Alterations in these hormones may contribute to tumorigenesis and invasion. Persistent insulin resistance enhances pancreatic insulin secretion, leading to hyperinsulinemia. Insulin-like growth factor I (IGF-I), produced by pancreatic beta cells like insulin, shares a similar molecular structure. The nuclear effects of IGF-1 correlate with cancer cell biology. Hyperlipidemia: A case-control study of 3,524 gallbladder cancer patients [16] indicated a significantly higher incidence of gallbladder cancer in patients with hypertriglyceridemia compared to those without (*P* < 0.001). Multivariate regression analysis suggested elevated serum triglyceride (TG) levels may be a predictive factor for gallbladder cancer risk, particularly in patients with gallstones. However, the mechanism by which elevated blood lipids contribute to gallbladder cancer development requires further investigation. The role of cholesterol levels in the progression of various cancers remains controversial. Hyperglycemia: Elevated blood glucose impairs gallbladder contractility, leading to bile stasis. This causes chronic inflammatory stimulation and oxidative damage to the gallbladder epithelium, potentially triggering carcinogenesis. Bile stasis may also exert carcinogenic effects by acting on the farnesoid X receptor (FXR), regulating bile acid homeostasis and tumor suppressor gene expression [17]. Reports also suggest elevated blood glucose may increase gallbladder cancer risk by promoting tumor growth, DNA damage, and altered RNA transcription. A nationwide cohort study revealed that among individuals with prediabetes at initial screening, persistent prediabetes significantly increased GBC risk, whereas remission from prediabetes did not elevate GBC risk, indicating that resolving prediabetes may be beneficial for reducing GBC risk [18].

### Other gallbladder lesions gallbladder polyps

The vast majority of small polyps are benign. However, polyps >10 mm in size, broad-based and sessile, solitary, associated with gallstones, or rapidly enlarging carry a significantly increased risk of malignancy. Current guidelines generally recommend prophylactic cholecystectomy for polyps >10 mm [19]. Pancreaticobiliary Malformation (PBM): PBM causes pancreatic juice reflux into the biliary tract, where activated pancreatic enzymes inflict persistent damage to the biliary mucosa, constituting a clear precancerous lesion [3]. “Porcelain” Gallbladder: Extensive calcification of the gallbladder wall is highly correlated with gallbladder cancer risk. While previously considered to carry an extremely high cancerization rate, recent studies have revised this assessment. It remains an indication for surgery [20]. Age, gender, geographic/ethnicity, long-term exposure to certain heavy metals (e.g., nickel, chromium, radon), primary sclerosing cholangitis, and a family history of gallstones are all risk factors for gallbladder cancer.

### Treatment progress and controversies

**Fig. 1:**
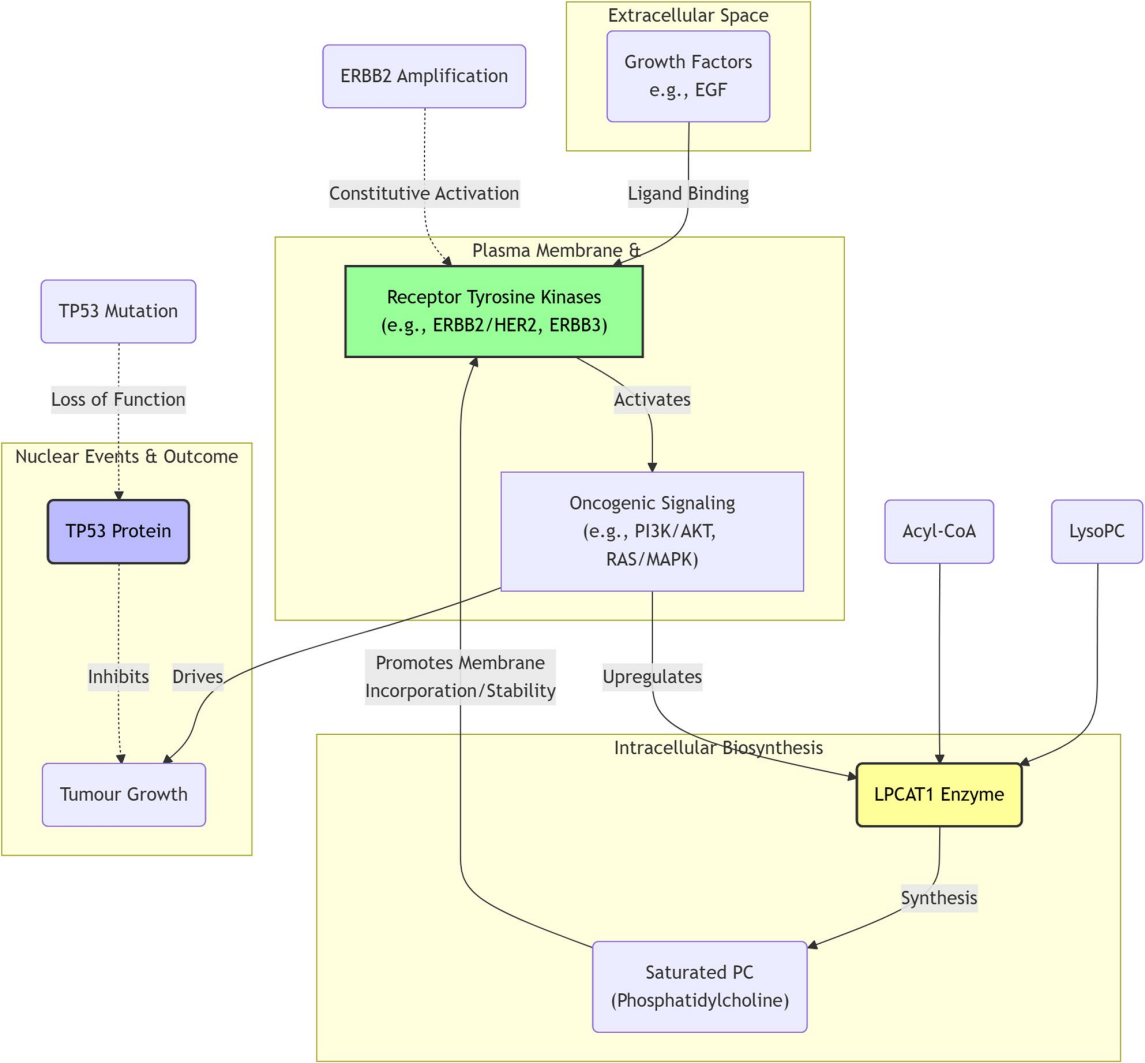
Partial molecular mechanisms of gallbladder cancer development

Surgery is the only potentially curative treatment for GBC. Treatment strategies are highly dependent on precise TNM staging (AJCC 8th edition) [21]. In recent years, surgical approaches have become increasingly individualized and precise, yet remain controversial on multiple fronts (Fig. 1).

#### Tis/T1a Stage GBC consensus

For in situ carcinoma (Tis) and tumors invading the lamina propria (T1a), simple cholecystectomy is sufficient, with a lymph node metastasis rate < 5% [22]. Lymph node dissection is not required, and laparoscopic surgery may be preferred. However, caution is warranted regarding implant metastasis caused by gallbladder rupture.

#### T1b/T2 stage GBC

For gallbladder cancer (GBC) involving the muscularis propria (T1b) and perimuscular connective tissue (T2), the risk of lymph node metastasis significantly increases, necessitating radical cholecystectomy (including cholecystectomy, partial hepatectomy, and regional lymph node dissection). However, specific surgical approach selection varies internationally. According to the Chinese Guidelines for Diagnosis and Treatment of Gallbladder Cancer (2019 Edition) [23], cholecystectomy combined with a hepatic wedge resection extending at least 2 cm beyond the gallbladder bed and regional lymph node dissection is recommended. However, some studies suggest that since hepatic wedge resection may not fully achieve R0 resection margins, cholecystectomy plus standard segmental resection (IVb + V) with regional lymph node dissection is safer for T1b patients [24]. Japanese guidelines, however, suggest that cholecystectomy alone may be sufficient unless intraoperative suspicion of lymph node metastasis arises [25].

#### T2 stage GBC

The 8th edition of the AJCC TNM staging system further subdivides stage T2 gallbladder cancer into stage T2a (tumor invades the peritoneal-facing periportal connective tissue but does not penetrate the serosa) and stage T2b (tumor invades the hepatic-facing periportal connective tissue but does not invade the liver). The extent of liver resection should be determined based on tumor location. Chinese guidelines for gallbladder cancer diagnosis and treatment recommend wedge resection of the liver (>2 cm) for tumors located on the peritoneal side (T2a). For tumors on the hepatic side (T2b), which are prone to invading the Glisson’s system and exhibit a higher incidence of nerve invasion, standard hepatectomy involving segments IVb and V is recommended [23]. A retrospective, multicenter propensity score-matched study demonstrated that compared to wedge resection, segmental resection of segments IVb and V in T2b gallbladder cancer did not improve overall survival but prolonged disease-free survival. However, segmental resection of segments IVb and V was associated with a higher incidence of postoperative complications (28.5% vs. 9.2%, *P* < 0.001) [26]. Our team recommends selecting segmental resection based on patient condition and surgeon proficiency. Laparoscopic radical cholecystectomy (LRRC) has gained prominence in recent years. Multiple retrospective studies indicate that in experienced centers, LRRC for selected T2-stage patients yields lower intraoperative bleeding, complication rates, and shorter hospital stays compared to open surgery, with no difference in short-term oncological outcomes [27]. However, concerns remain regarding long-term oncological safety and port site implantations. The 2023 Expert Consensus on Laparoscopic Radical Cholecystectomy for Gallbladder Cancer recommends strict case selection for LRRC (e.g., non-ulcerated tumors, no distant metastasis) and execution by technically proficient teams to ensure lymph node dissection quality and R0 resection rates [28]. Extrahepatic Biliary Duct Resection (EHBD): Routine resection is not recommended. It is indicated only for patients with positive gallbladder duct margins or direct tumor invasion of the extrahepatic bile ducts, as it offers no survival benefit but significantly increases the risk of surgical complications (e.g., bile leakage, stricture) [23, 25].

#### T3/T4 stage GBC

Treatment strategies become more complex for T3 (tumor penetrating the gallbladder serosal layer and/or directly invading the liver and/or one adjacent organ) and T4 (tumor invading the portal vein or hepatic artery, or two or more extrahepatic organs) lesions. Regarding surgical approach selection, standard radical resection typically involves cholecystectomy combined with partial hepatectomy (usually segments IVb and V) and regional lymph node dissection [29]. However, for more advanced disease, extended radical resection may be required, potentially encompassing right hepatectomy, right trisegmentectomy, combined extrahepatic choledochotomy, portal vein resection with anastomotic reconstruction, or even hepatopancreatoduodenectomy (HPD). It is noteworthy that different medical centers define surgical scope differently, reflecting the current diversity of concepts and technical disparities in gallbladder cancer treatment. Regarding T3 gallbladder cancer, opinions remain divided on whether extended radical resection confers survival benefits. Some early studies showed significantly higher 5-year survival rates in T3 gallbladder cancer patients undergoing extended radical resection compared to those receiving simple cholecystectomy. However, a Japanese meta-analysis [30] contradicted this, indicating that combined segmental resection of segments IVb and V of the liver did not improve postoperative liver metastasis rates, disease-free survival, or overall survival in T3 gallbladder cancer patients. Instead, it significantly increased postoperative complications. This conflicting evidence poses substantial challenges for surgeons in decision-making. For T4 gallbladder cancer, the controversy is even more pronounced. While Japanese reports [31] suggest that extended radical resection may prolong survival in patients with stage IV or higher advanced gallbladder cancer who tolerate surgery, even with lymph node or liver metastases, opposing views emphasize the procedure’s extensive scale, high risk, high complication rate, significant cost, and elevated postoperative mortality. Of particular concern is the high complication rate (35%−100%) and mortality (0–47%) associated with hepatopancreaticoduodenectomy (HPD), one of the most complex radical resections. Given these risks, many scholars question HPD’s value in gallbladder cancer treatment, especially considering the grim 3-year overall survival rate of only 4% for T4 patients [32] [32]. Another core controversy lies in the lack of clear patient selection criteria. Currently, no unified standards exist to determine which T3/T4 gallbladder cancer patients are most suitable for extended radical resection. The extent of lymph node dissection is another point of contention. While current consensus recommends obtaining at least six lymph nodes for accurate staging, the impact of dissection scope on treatment outcomes remains unclear [33]. The CSCO Expert Consensus on Diagnosis and Treatment of Biliary Tract Tumors (2019 Edition) [29] recommends extending dissection to perihilar lymph nodes if intraoperative No. 8 or No. 13 nodes are positive. However, positive para-aortic lymph nodes (No. 16 nodes) are typically regarded as distant metastasis with poor prognosis and are generally not considered for surgical intervention. These uncertainties allow considerable flexibility in lymph node dissection strategies for gallbladder cancer, further highlighting the need for more precise guidelines. Regarding neoadjuvant therapy, while it has established roles in other gastrointestinal cancers, its application in gallbladder cancer—particularly for T3/T4 stage patients—remains highly controversial. On one hand, studies suggest neoadjuvant therapy may induce tumor downstaging and improve R0 resection rates [34]; on the other hand, most current studies are small-sample retrospective analyses lacking high-quality prospective randomized controlled trial evidence. Furthermore, the unclear conceptual distinction between neoadjuvant and conversion therapy adds to the debate. Academically, neoadjuvant therapy targets initially operable patients, while conversion therapy targets initially inoperable patients—fundamentally differing in treatment goals and patient selection. This conceptual confusion may compromise clinical study accuracy and treatment strategy optimization. Furthermore, there remains disagreement regarding whether extrahepatic choledochotomy should be routinely incorporated into radical resection for gallbladder cancer. Early studies demonstrated that combined extrahepatic choledochotomy significantly improved outcomes in patients with gallbladder cancer involving nerve invasion, leading to recommendations for routine extrahepatic choledochotomy in radical surgery for stage T2 or higher gallbladder cancer. However, multiple recent clinical studies have confirmed that for patients with non-invasive cholangiocarcinoma, extrahepatic cholangiectomy does not improve prognosis or quality of life. Instead, it prolongs operative time and increases the incidence of complications such as intraoperative hemorrhage and postoperative bile leakage [35]. This divergence underscores the importance of individualized decision-making in gallbladder cancer treatment and highlights the current limitations in the evidence base. These controversies not only reflect the complexity of managing gallbladder cancer but also emphasize the urgent need for high-quality research to address gaps in the evidence. In light of these debates, the multidisciplinary team (MDT) collaboration model becomes particularly crucial, enabling the provision of more personalized and comprehensive treatment plans for patients.

#### Systemic therapy for advanced and recurrent gallbladder cancer

For patients who are inoperable or have recurrent disease, systemic therapy is central. Traditional chemotherapy with gemcitabine plus cisplatin (GC) has limited efficacy. Immunotherapy combining checkpoint inhibitors with chemotherapy has become the standard first-line treatment, significantly improving patient survival [36]. The Phase III TOPAZ-1 study demonstrated that durvalumab (a PD-L1 checkpoint inhibitor) combined with GC treatment for advanced GBC yielded superior overall survival compared to GC alone [37]– [38]. Molecularly targeted precision therapy has emerged as a research frontier: Pemetrexed is recommended for patients with FGFR2 fusions/rearrangements; Encoritinib or Larotrectinib for NTRK fusion-positive cases; Ivosidenib for IDH1 mutations; Dabrafenib plus Trametinib for BRAF V600E mutations; HER2-positive patients may opt for pertuzumab plus trastuzumab or tixotuzumab; RET fusion patients are recommended pralsetinib or seputinib; NRG1 fusion patients are recommended zatuzumab. These advances are progressively reshaping the treatment landscape for advanced GBC, and future exploration of combination regimens with surgery and radiotherapy warrants investigation.

## Summary and outlook

This paper provides a systematic review of risk factors and surgical treatment advances for gallbladder cancer (GBC). The prevention and treatment of GBC constitute a systematic endeavor. Future efforts should focus on the following areas: Risk Stratification and Early Diagnosis: Establish predictive models integrating molecular biomarkers (e.g., ctDNA), imaging characteristics, and clinical risk factors to enable precise screening and early diagnosis in high-risk populations. Standardization and Minimally Invasive Surgery: Prospective, multicenter randomized controlled trials are urgently needed to resolve controversies regarding surgical extent for T1b/T2 stages and laparoscopic application with high-level evidence, thereby advancing standardized and minimally invasive surgical practices. Multimodal Integrated Therapy: Actively explore combined approaches integrating neoadjuvant/adjuvant therapy with surgery. Immunotherapy and targeted therapy hold broad application prospects, requiring clarification of optimal patient populations and timing for administration. Basic and Translational Research: Deepen understanding of the molecular mechanisms underlying GBC development, particularly the immunosuppressive characteristics of the tumor microenvironment (TME), to provide theoretical foundations for identifying novel therapeutic targets. In summary, confronting the challenge of GBC requires a multifaceted approach encompassing prevention, early screening, precise staging, individualized surgery, and multidisciplinary comprehensive treatment to ultimately improve patient prognosis.

## Data Availability

No datasets were generated or analysed during the current study.
